# Ruthenium Incorporated Cobalt Phosphide Nanocubes Derived From a Prussian Blue Analog for Enhanced Hydrogen Evolution

**DOI:** 10.3389/fchem.2018.00521

**Published:** 2018-10-30

**Authors:** Yingzhang Yan, Jinzhen Huang, Xianjie Wang, Tangling Gao, Yumin Zhang, Tai Yao, Bo Song

**Affiliations:** ^1^Department of Physics, Harbin Institute of Technology, Harbin, China; ^2^Centre for Composite Materials and Structures, Harbin Institute of Technology, Harbin, China; ^3^Institute of Petrochemistry, Heilongjiang Academy of Sciences, Harbin, China; ^4^Academy of Fundamental and Interdisciplinary Sciences, Harbin Institute of Technology, Harbin, China

**Keywords:** hydrogen evolution reaction, metal–organic framework, prussian blue analog, nanocube, electrocatalysis

## Abstract

Electrochemical water splitting in alkaline media plays an important role in mass production of hydrogen. Ruthenium (Ru), as the cheapest member of platinum-group metals, has attracted much attention, and the incorporation of trace amount of Ru with cobalt phosphide could significantly improve the hydrogen evolution reaction (HER) catalytic activity. In this work, ruthenium-incorporated cobalt phosphide nanocubes are synthesized via a reaction between Co–Co Prussian blue analog (Co-PBA) and ruthenium chloride (RuCl_3_) followed by the phosphidation. The sample with a Ru content of ~2.04 wt.% exhibits the best HER catalytic activity with a low overpotential of 51 and 155 mV, to achieve the current densities of −10 and −100 mA cm^−2^, respectively, and the Tafel slope of 53.8 mV dec^−1^, which is comparable to the commercial Pt/C. This study provides a new perspective to the design and construction of high performance electrocatalysts for HER and other catalytic applications in a relatively low price.

## Introduction

Electrochemical water splitting is considered as one of the most promising methods for massive hydrogen production (Zou and Zhang, 2015; Roger et al., 2017). However, large overpotential caused by sluggish kinetics of hydrogen evolution reaction (HER) and oxygen evolution reaction (OER), to a large extent, reduced the electrical conversion efficiency. By far, great efforts have been devoted to searching more efficient electrode catalysts that could accelerate the HER kinetics (Xiao et al., 2016). Platinum (Pt)-based catalysts are still the state of the art with extremely low overpotential and excellent long-term durability (Shi and Zhang, 2016), but the high price and scarcity hinder their practical application in large scale. To address this issue, an alternative with non- or low noble metal content is currently in great demand. As a result, in the past few years, considerable efforts have been dedicated to exploring earth-abundant electrocatalysts based on first-row (3d) transition metals (Wan et al., 2014; Anantharaj et al., 2016; Song et al., 2017).

Among those as-synthesized nonprecious metal-based electrocatalysts, transition-metal phosphides (TMPs) stood out as a group of promising catalysts for HER (Shi and Zhang, 2016), in which the negatively charged nonmetal atoms and isolated metal atoms functioned as proton-acceptor sites and hydride-acceptor, respectively. The co-existence of proton-acceptor sites and hydride-acceptor as well as the excellent conductivity would facilitate the electrocatalytic process (Zou and Zhang, 2015). Cobalt phosphide (CoP), as a representative of TMPs, has been demonstrated to show great catalytic activity toward HER. Popczun et al. (2014) synthesized multi-faceted, hollow CoP nanoparticle as a high active HER catalyst. After that, various CoP nanostructures such as nanowires (Jiang et al., 2014; Tian et al., 2014), hierarchical nanostructures (Popczun et al., 2015), nanoneedle array (Fu et al., 2018), etc. were investigated toward HER.

However, restricted by the intrinsic Gibbs free energy of hydrogen adsorption (Δ${\text{G}}_{\text{H}}^{0}$), the catalytic ability of CoP is still inferior to that of Pt-based catalyst, and it is hard to further enhance the HER performance via the structure designing and optimization. It was shown that doping element into the matrix of CoP could improve their catalytic activity since the introduction of proper elements could (i) modulate the Δ${\text{G}}_{\text{H}}^{0}$ thus improve the electrochemical reaction kinetics, (ii) lead to the formation of lattice defects, so that more electrons will be offered to P atoms for enhanced activity.(Jin et al., 2014) Plenty of works have been done about effect of doping elements such as Al (Zhang et al., 2017), Ni (Feng et al., 2016; Han et al., 2017), Fe (Tang et al., 2016), Ce (Gao et al., 2017; Zhang et al., 2018), and Mn (Liu et al., 2016) on the catalytic properties of CoP. Recently, Xu et al. (2018) reported that the introduction of Ru into CoP could efficiently reduce the binding energy of H_ads_, thus promise its Pt-like HER catalytic ability by using the density functional theory (DFT) calculations. However, how to balance the catalytic performance and the cost of the catalyst, i.e., to search a low-Ru content cobalt phosphide catalyst with high performance, has yet to be performed.

Herein, we synthesized a low Ru content (2.04 wt.%) CoP nanocubes using Co-based Prussian Blue Analog (Co-PBA, Co_3_(Co(CN)_6_)_2_) as the precursor. The Ru-doped Co-PBA was prepared via an ion-exchange reaction between Co-PBA and RuCl_3_, and the content of Ru could be facilely adjusted by varying the concentration of RuCl_3_. The Ru incorporated CoP (Ru-CoP) was then synthesized through a facile phosphorization at 350°C using NaH_2_PO_2_ as phosphorus source. By using this pathway, Ru could be evenly distributed in the Co-PBA, and the derived Ru-CoP with a porous and rough morphology features, but still remaining a cubic architecture after the low-temperature phosphidation process were achieved. Note that, the Ru-CoP could be facilely synthesized on the gram scale, which is advantageous for practical applications.

The optimal Ru-CoP nanocubes deposited on a glassy carbon electrode (GCE) exhibits excellent HER catalytic activity with a low overpotential of 51 and 155 mV, to achieve the current densities of −10 and −100 mA cm^−2^, respectively, and the Tafel slope of 53.8 mV dec^−1^, which is even comparable to that of commercial Pt/C. This work provides a new perspective to enhance the HER electrocatalytic performance of CoP-based catalyst, which not only enables us to achieve superior HER performance via a simple ion-exchange reaction followed by a phosphidation process, but also paves the way for further designing and construction of high performance electrocatalysts and other catalytic applications in a relatively low price.

## Experimental

### Catalysis synthesis

Ru-incorporated CoP was synthesized via the reaction between Co-PBA and RuCl_3_ followed by the phosphidation treatment. Specifically, Co_3_(Co(CN)_6_)_2_ was synthesized via a coprecipitation process according to the reference (Su et al., 2017). Solution A was composed of 6 mmol CoCl_2_·6H_2_O and 9 mmol trisodum citrate in 100 mL ultrapure water (18.25 MΩ), Solution B was made up of 4 mmol K_3_(Co(CN)_6_) in 100 mL ultrapure water. Solution A was mixed with solution B under ultrasonic treatment for 1 h. Then, the obtained suspension liquid was kept still overnight for aging and precipitating. The Co-PBA was collected by centrifugation, washed with distilled water and ethanol repeatedly over three times, and finally dried under 80°C for 12 h. To synthesize Ru-Co-PBA-1, 500 mg as-prepared Co-PBA were first re-dispersed in 150 mL ultrapure water, and then mixed with 50 mL solution that contains 70 mg RuCl_3_ under ultrasonic treatment for 10 h in dark. The product was then collected by centrifugation. Ru-Co-PBA-2 was also synthesized via similar steps, except that the content of RuCl_3_ was changed to 140 mg. To synthesize Ru-CoP-1-350, the obtained brown-colored precursor Ru-Co-PBA-1 together with NaH_2_PO_2_ were placed side by side in a tube furnace at the mass ratio of 1:10, while NaH_2_PO_2_ was placed at the upstream side. The phosphating temperature was raised to 350°C with a heating rate of 2°C min^−1^ and kept for 2 h under 100 sccm high-purity argon (99.999%) flow. CoP-350, Ru-CoP-2-350 were also synthesized by the same steps using Co-PBA and Ru-Co-PBA-2 as precursors, respectively.

### Characterization

Powder X-ray diffraction (XRD) patterns of the obtained PBA precursors and catalysts were detected with an X'Pert Pro diffractometer at a scan rate of 10° min^−1^. Scanning electron microscopy (SEM) images were collected by a Hitachi S-4700 microscope. Transmission electron microscopy (TEM) images and energy dispersive spectroscopy (EDS) were collected using a Tecnai G2 F20 S-TWIN microscope. X-ray photoelectron spectroscopy (XPS) data were obtained using an ESCALAB KII spectrometer with Al Kα as the excitation source. The specific surface and pore diameters were obtained from the results of N_2_ physisorption at 77 K (ASAP2460) using the Brunauer-Emmet-Teller (BET) method.

### HER electrochemical measurement

The HER electrochemical measurements were performed on an electrochemical workstation (CH 760E) in 1M KOH electrolyte using three-electrode system. In a typical process, 5 mg of catalyst and 50 μL Nafion solution (Alfa Aesar, 5 wt.%) were dispersed in 500 μL of ethanol to form a homogeneous ink. Then, 7 μL of the dispersion was loaded onto a glassy carbon electrode (GCE) with 5 mm diameter (loading 0.3 mg cm^−2^) to obtain the working electrode. An HgO/Hg electrode and a graphite electrode were served as the reference electrode and counter electrode, respectively. All of the potentials mentioned later were calibrated to reversible hydrogen electrode (RHE). Linear sweep voltammetry (LSV) with a scan rate of 10 mV s^−1^ was conducted between−0.6 and 0 V vs. RHE in 1M KOH electrolyte. Electrochemical impedance spectroscopy (EIS) was conducted in the frequency range from 100 kHz to 0.01 Hz with an ac perturbation of 5 mV at the potential of −0.1 V vs. RHE. Cyclic voltammetry (CV) was conducted with scan rate from 20 to 200 mV s^−1^ ranging from 0.05 to 0.15 V vs. RHE. In order to prevent the mass loss cause by the drastic gas evolution, the stability test was performed using carbon paper (5 × 5 mm, Toray Industries, INC., Japan) as the substrate with catalyst loading of 0.3 mg cm^−2^ at an electric potential of −0.08 V vs. RHE for over 50 h.

## Result and discussion

**Figure 1:**
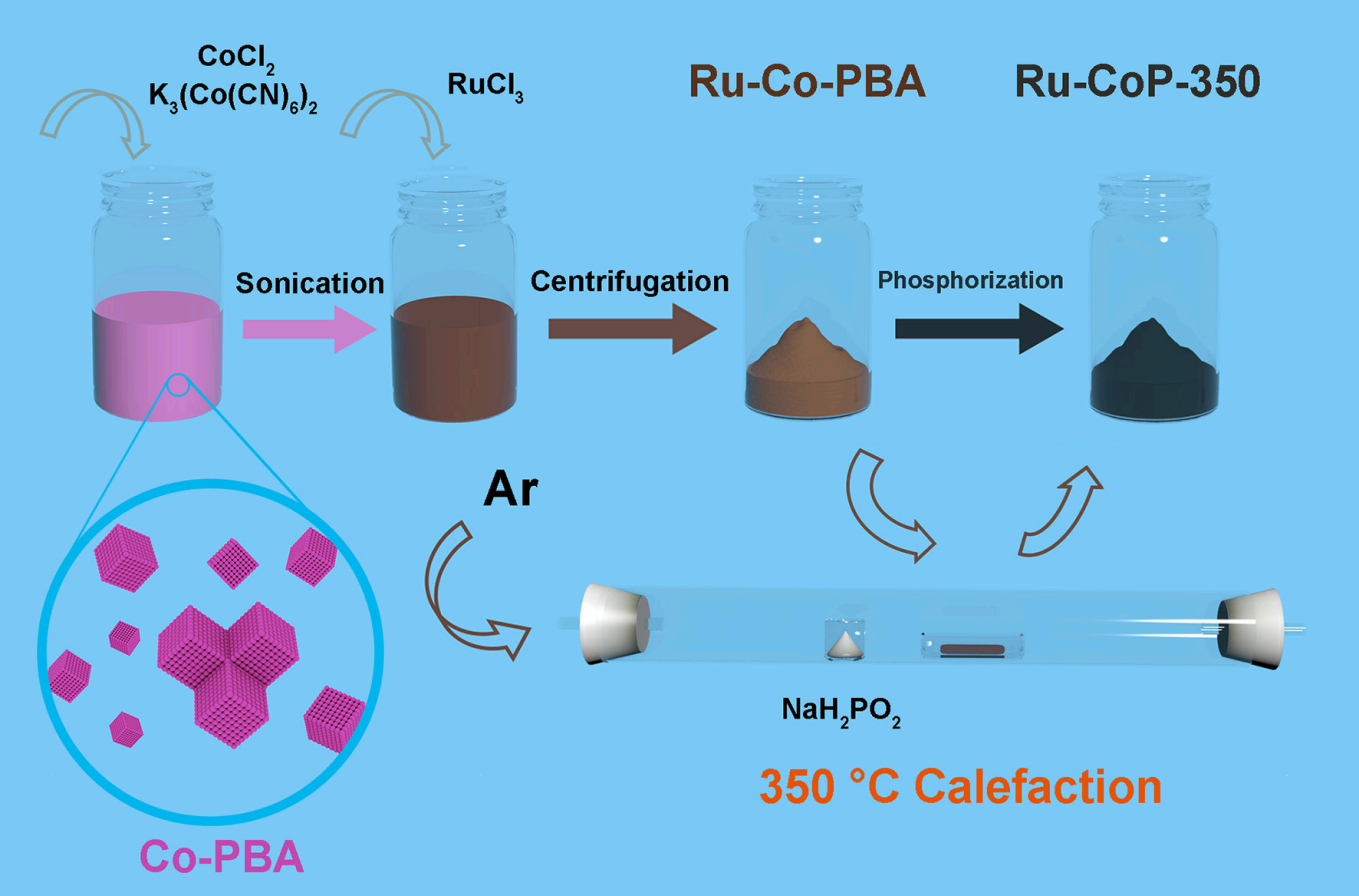
Schematic illustration for the synthesis of Ru-CoP-350 catalyst.

**Figure 2:**
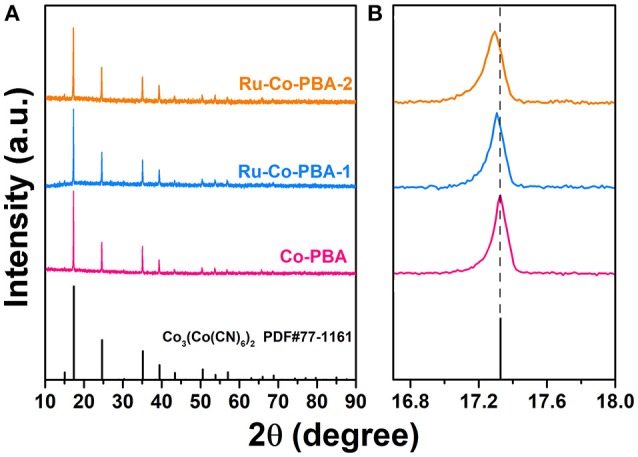
XRD patterns of **(A)** Co-PBA, Ru-Co-PBA-1 and Ru-Co-PBA-2 samples and **(B)** the magnified region of 2θ = 16.7–18.0° showing a gradual peak shift.

The Schematic illustration showing the synthetic route of the Ru-CoP particles is displayed in Figure 1. Co-PBA nanocudes were synthesized through coprecipitation of CoCl_2_ and K_3_(Co(CN)_6_)_2_, then the Ru-doped Co-PBA nanocubes were obtained via ion-exchange reaction between Co-PBA and RuCl_3_ solution (Su et al., 2017). XRD patterns were collected to determine the crystal structure of the precursor (Figure 2). All the PBA samples are highly crystalline and the introduction of Ru ion does not change the crystal structures, since Ru-Co-PBA samples still maintain the face-centered cubic structure of Co_3_[Co(CN)_6_]_2_ (JCPDS 77-1161). A closer look at the main (200) peak in the 2θ range of 16.7–18.0° (Figure 2B) reveals a gradual shift to lower angle with increased Ru content, caused by the expansion of lattice constant when the Co ions (~0.58 Å for Co^3+^ and 0.65 Å for Co^2+^) are partially substituted by Ru ions with a larger radius (~0.68 Å for Ru^3+^) (Song et al., 2017). Ru-CoP-350 was prepared by phosphating the Ru-doped Co-PBA nanocubes under Ar atmosphere. Supplementary Figure 1 (in the Supplementary Material) shows the XRD patterns of the Ru-CoP. It is found that characteristic diffraction peaks of Co_3_[Co(CN)_6_]_2_ disappeared after phosphidation. The broad humps are well accordant with characteristic diffraction peaks of CoP (JCPDS 89-2747), indicating the successful synthesis of CoP.

**Figure 3:**
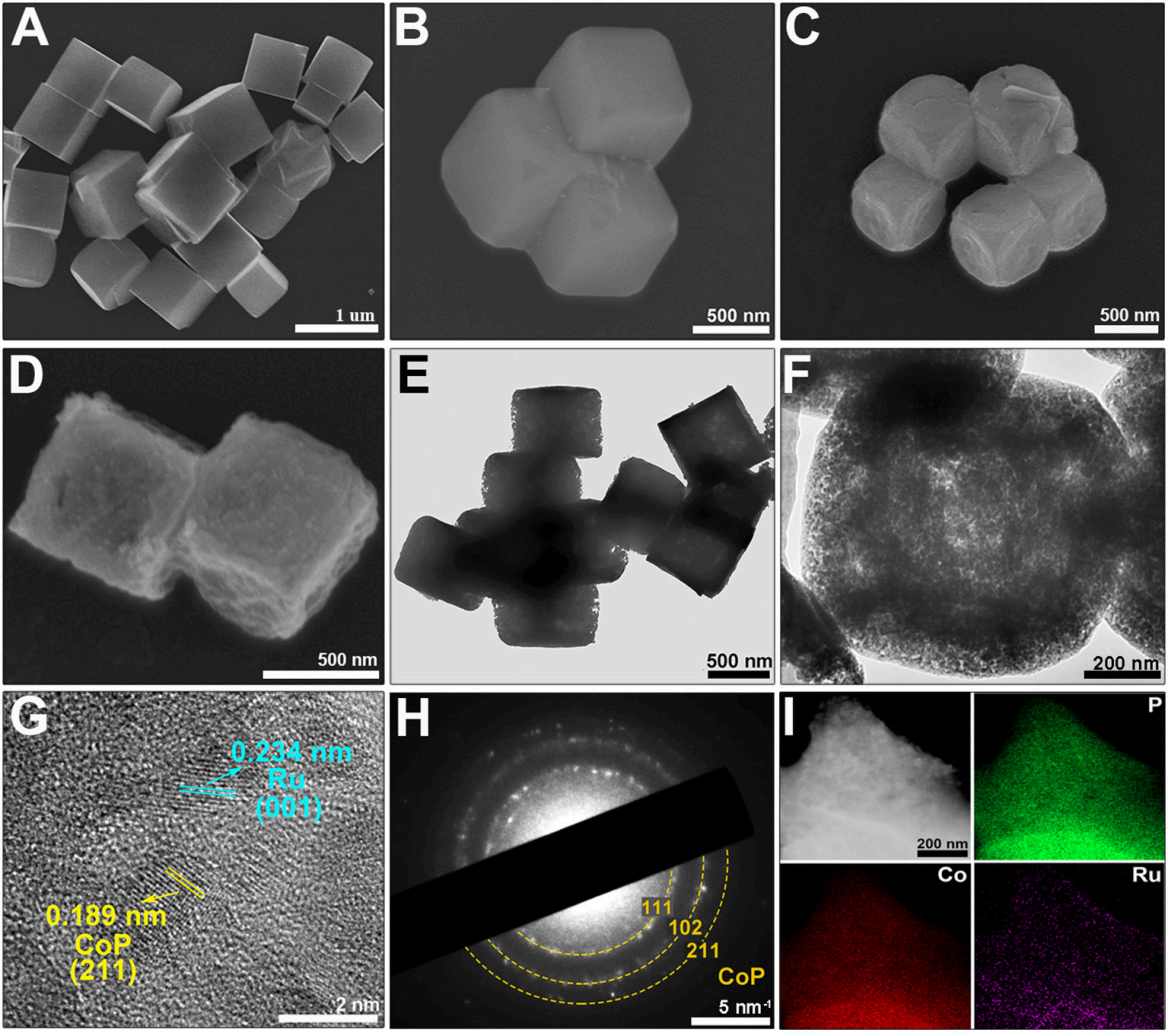
SEM images of **(A,B)** Co-PBA, **(C)** Ru-Co-PBA-2 and **(D)** Ru-CoP-2-350. **(E,F)** TEM images, **(G)** HRTEM image, **(H)** SAED and **(I)** elemental mapping (P, Co, Ru) of Ru-CoP-2-350.

According to the SEM results, the Co-PBA present a homogeneous cubic morphology with a size of around 500 nm, and their surface are relatively smooth (Figures 3A,B). The Ru-doped Co-PBA basically inherit the cubic architecture and size of the Co-PBA, but with rough surface due to the expansion of lattice constant caused by the ion-exchange reaction (Figure 3C). During the phosphating process at 350°C, PH_3_ produced from the decomposition of the NaH_2_PO_2_ served as the phosphorus source and reacted with the Ru-doped Co-PBA, which further increased the roughness of the particle surface (Figure 3D). The specific surface area and pore size distribution of Ru-CoP-2-350 were obtained by N_2_ adsorption/desorption isotherms. As can be seen in Supplementary Figure 2, a type-II isotherm with a H3-type hysteresis loop is obtained, which is characteristic of mesoporous non-rigid aggregates (Su et al., 2017). The specific surface area obtained by the BET method is 2.64 m^2^ g^−1^. The internal porous structure of the nanocubes can be clearly observed through the TEM images (Figures 3E,F), which can enlarge the specific surface area and help to expose more active sites. From the HRTEM image of Ru-CoP-2-350 (Figure 3G), lattice interplanar spacing of 0.189, and 0.234 nm can be identified, which are well consistent with the (211) plane of the orthorhombic CoP phase (JCPDS No: 89-2747), and (001) plane of the hexagonal Ru phase (JCPDS No: 06-0663), respectively. The existence of Ru^0^ is mainly due to the strong reductibility of PH_3_ which could reduce Ru^3+^ into Ru^0^, since the formation of RuP requires an even tougher reaction condition (Pu et al., 2017). Selected area electron diffraction (SAED) shows that the nanoparticles adopt the pattern expected for CoP (Figure 3H) (Popczun et al., 2014), and the diffraction rings reveals the polycrystalline character of the material, which is well corresponding to the result of XRD (Supplementary Figure 1). The elemental mappings of Ru-CoP-2-350 (Figure 3I) reveal a homogeneous distribution of P, Co, Ru throughout the nanocube, further indicating that Ru is well incorporated with CoP. The Ru content of Ru-CoP-2-350 is 2.04 wt.% according to the EDS result (Supplementary Figure 3), which is well consistent with previous research (Su et al., 2017).

**Figure 4:**
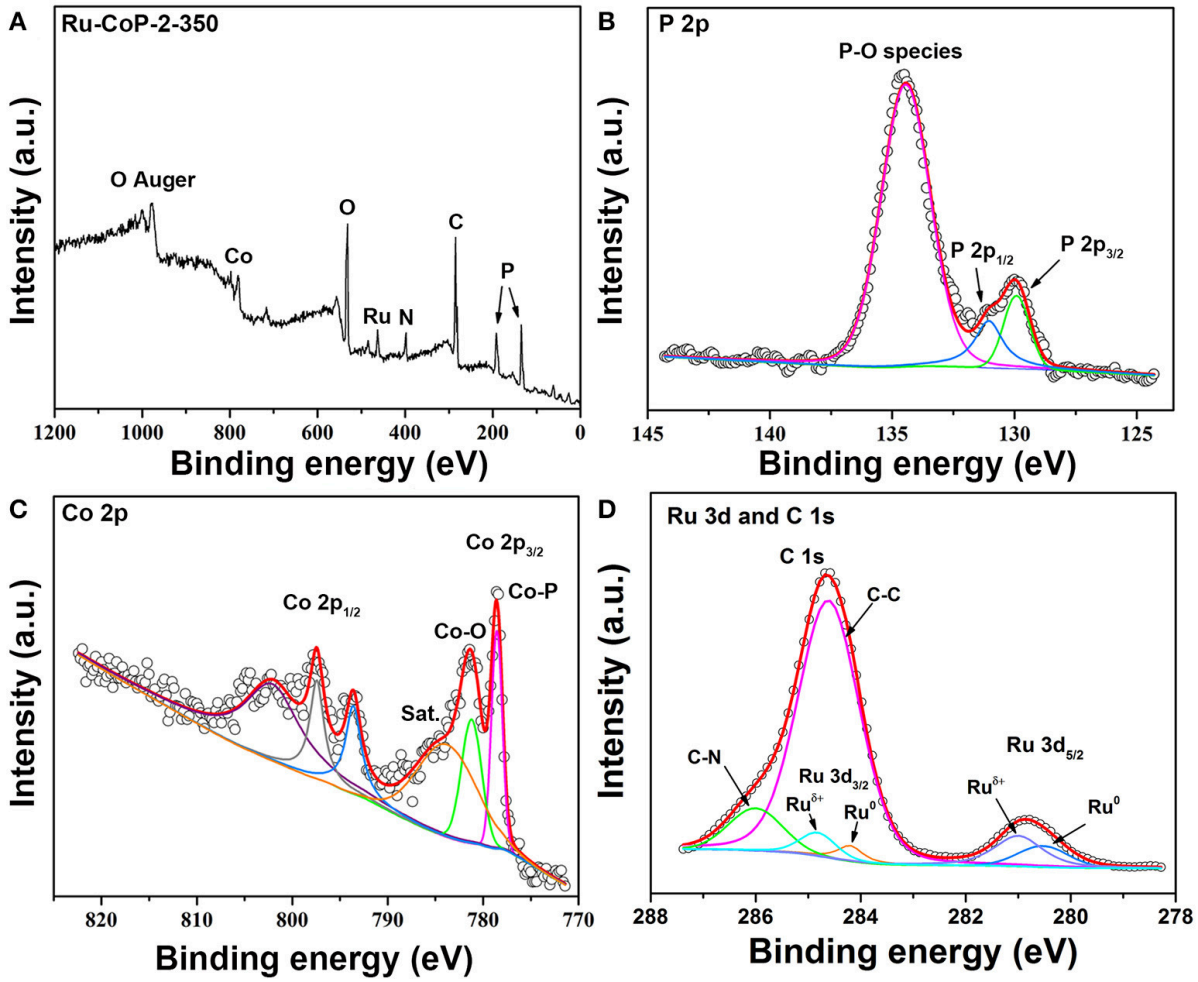
XPS spectra of Ru-CoP-2-350. **(A)** Full spectrum, **(B)** P 2p, **(C)** Co 2p, and **(D)** Ru 3d and C 1s XPS spectra.

To further analyze the element composition and valence states on the Ru-CoP-350 particle surface, X-ray photoelectron spectroscopy (XPS) detection was conducted on Ru-CoP-2-350 (Figure 4), and the full spectrum in Figure 4A confirms the existence of Co, P, and Ru. Figure 4B gives the high-resolution spectrum about P 2p, which could be divided into three peaks, two peaks located at 129.9 and 131.0 eV correspond to metal phosphides, and the other peak at 134.3 eV reflecting P–O bonding generated on the surface of Ru-CoP-350 due to the surface oxidation (Xuan et al., 2017). The Co 2p spectrum (Figure 4C) is splitted into 2p_3/2_ and 2p_1/2_ doublets due to spin orbit coupling. The spectrum revealed that cobalt exists as Co-P and oxidized Co species (Pan et al., 2016). High resolution spectrum of C 1s and Ru 3d is presented in Figure 4D. The C 1s was splitted into two peaks centered at 284.8 and 286.0 eV, which correspond to the C-C and C-N bonds, respectively. The Ru 3d spectrum is splitted into Ru 3d_5/2_ and Ru 3d_3/2_, and the peaks at 280.2 and 281.1 eV for Ru 3d_5/2_ were assigned to Ru^0^ and the Ru^δ+^ from RuO_x_ species (Cao et al., 2017; Xu et al., 2017).

**Figure 5:**
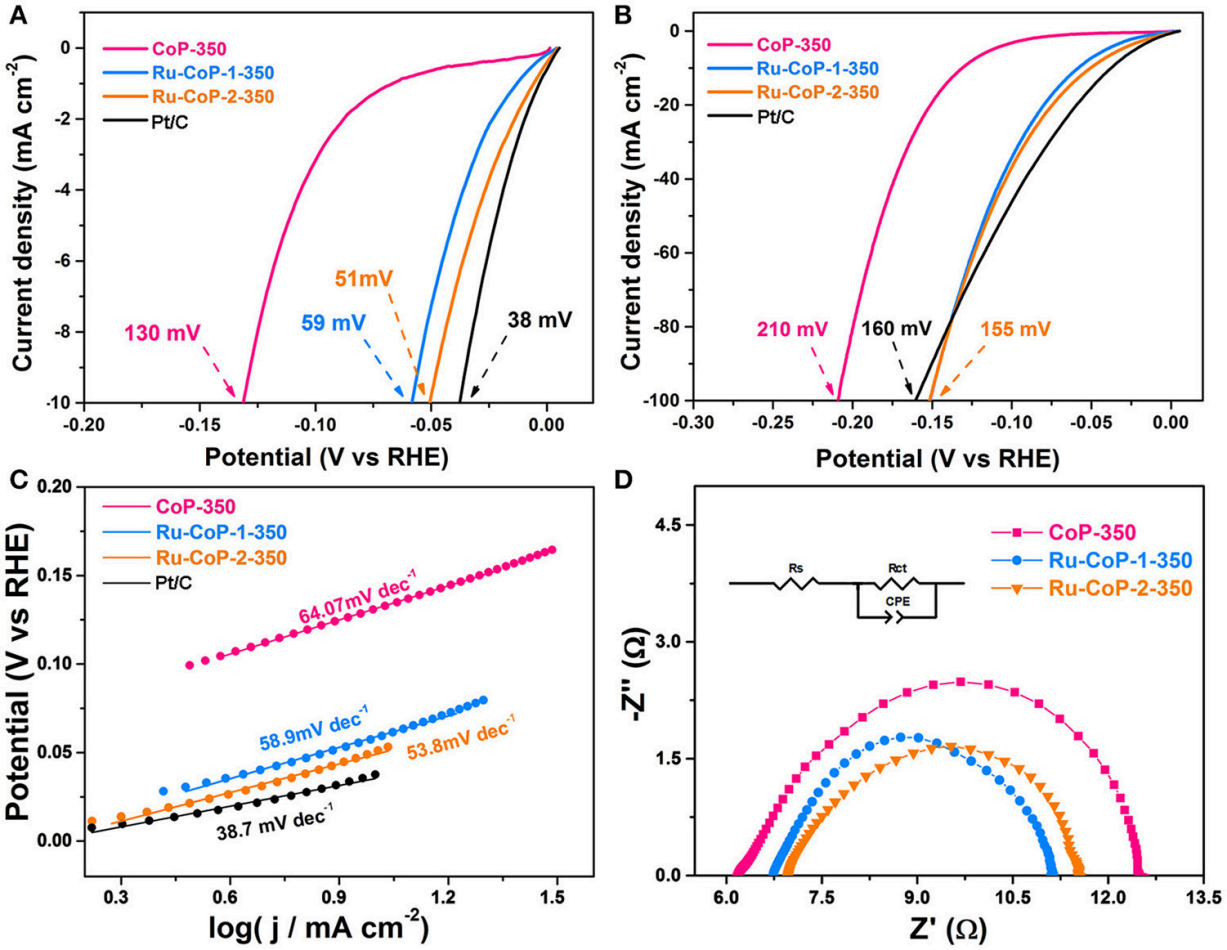
Electrochemical properties of CoP-350, Ru-CoP-1-350, Ru-CoP-2-350 and Pt/C for the HER in 1 M KOH. **(A,B)** HER polarization curves, **(C)** Tafel plots, **(D)** Nyquist plots. All measurements were carried out with a fixed catalyst loading of ~0.3 mg cm^–2^ on a GCE.

The HER electrocatalytic activity of CoP-350, Ru-CoP-350-1, Ru-CoP-350-2 were investigated in 1 mol L^−1^ KOH solution using a three-electrode setup with the catalyst-modified GCE as the working electrode. We note that, in compare of the primary undoped CoP materials, the doped one has apparent improvement in performance and electrochemical characteristics. As shown in Figures 5A,B, for Ru-CoP-2-350, the overpotentials of only 51 and 155 mV were required to achieve the current density of −10 and −100 mA cm^2^, respectively, which were much lower than those of CoP-350 (130 and 210 mV). This is also among the top performance of metal phosphides (Table S1). From the extrapolation of the linear region of overpotential vs. log *j* (Figure 5C), Tafel slopes of 64.07, 58.9, 53.8 mV dec^−1^ can be obtained for CoP-350, Ru-CoP-1-350 and Ru-CoP-2-350, respectively. An exceptionally low Tafel slope value of 53.8 mV dec^−1^ for Ru-CoP-2-350 suggests highly efficient kinetics for H_2_ evolution. To provide further insight into the electrode kinetics during HER catalysis, electrochemical impedance spectroscopy (EIS) were applied. The Nyquist plots (Figure 5D) display a “semicircle” feature which can be fitted using an equivalent circuit (inset in Figure 5D) to extract the charge transfer resistance (R_ct_) to be 6.47, 4.40, 4.56 Ω for CoP-350, Ru-CoP-1-350 and Ru-CoP-2-350, respectively. This results confirm that the ruthenium incorporation could improve the HER kinetics of the CoP sample.

**Figure 6:**
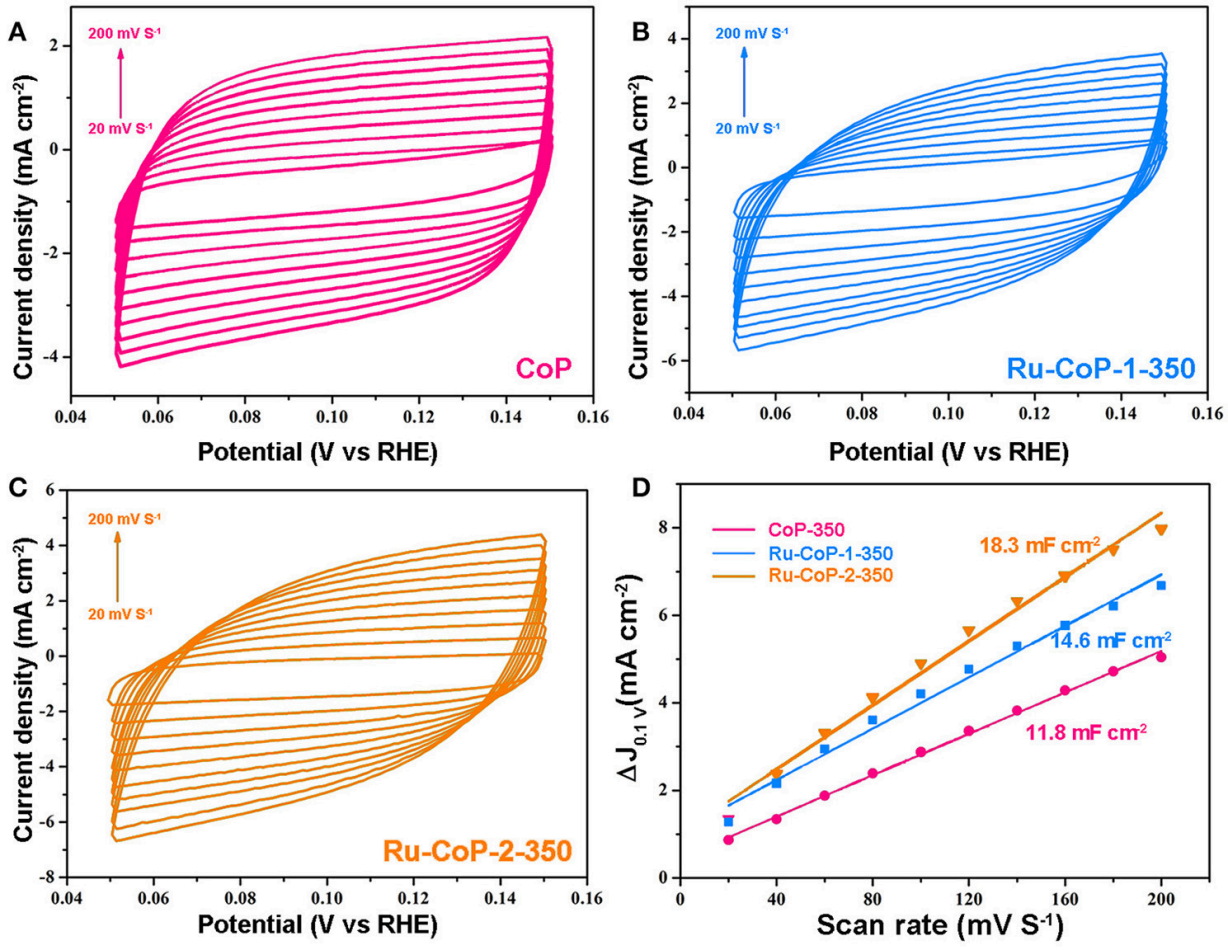
CV curves of scan rate ranging from 20 to 200 mV s^1^ for **(A)** CoP-350, **(B)** Ru-CoP-1-350, **(C)** Ru-CoP-2-350, **(D)** *C*_dl_ values of CoP-350, Ru-CoP-1-350, Ru-CoP-2-350.

Considering that all the samples have roughly the same physical particle diameter, the double-layer capacitance (*C*_dl_) is estimated from the cyclic voltammetry (CV) curves (Figures 6A–C for CoP-350, Ru-CoP-1-350 and Ru-CoP-2-350, respectively) to probe the effective electrochemically active surface area (ECSA) during HER catalysis. Capacitive current density was plotted as a function of scan rate to extract the *C*_dl_ values (Figure 6D) to be 11.8, 14.6, 18.3 mF cm^−2^ for CoP-350, Ru-CoP-1-350 and Ru-CoP-2-350, respectively. This suggests that Ru-incorporated CoP samples display more catalytically active sites in comparison to the undoped CoP sample. To compare the intrinsic catalytic activity of the samples, the turnover frequency (TOF) were calculated. The Ru-CoP-2-350 achieves the TOF value of 0.15 s^−1^ at the overpotential of 100 mV, which is much better than that of CoP-350 (0.02 s^−1^), indicating that Ru-CoP-2-350 has a better intrinsic catalytic activity. The calculation detail was provided in the Supplementary Material (results is in Supplementary Figure 4). Electrochemical durability is also important for the electrocatalysts in practical applications. The durability test of the best-performed Ru-CoP-2-350 catalyst was conducted at the constant potential (−0.08 V vs. RHE). The catalytic current density remains stable at around 10 mA cm^−2^ for the HER after 50 h (Supplementary Figure 5), and the morphology of the catalyst after stability test remains a cubic architecture (Supplementary Figure 6), confirming that Ru-CoP-2-350 is a promising and stable HER electrocatalyst.

## Conclusion

In summary, we have successfully synthesized Ru-incorporated CoP nanocubes. The catalyst basically inherit the cubic structure of Co-PBA, and the porous and rough morphology caused by the phosphorization further enlarge its specific surface area. Electrochemical characterization demonstrates that Ru-CoP-2-350 with a Ru content of 3.66 atom%, exhibits the best HER activities with low overpotentials of 51 and 155 mV, to achieve the current densities of −10 and −100 mA cm^−2^, respectively, and the Tafel slope of 53.8 mV dec^−1^, which is even comparable to those that of commercial Pt/C. This work enables us to achieve superior HER performance via a simple ion-exchange reaction followed by a phosphidation process, and provide a new option to develop a high-performance HER electrocatalysts in alkaline media with less noble metal consumption.

## Author contributions

YY partly designed the experiments and wrote the manuscript. JH, XW, and YZ assisted in the analysis and interpretation of the data. TG, TY, and BS proposed the project, designed the experiment, and wrote the manuscript.

### Conflict of interest statement

The authors declare that the research was conducted in the absence of any commercial or financial relationships that could be construed as a potential conflict of interest.
