# Supplementary material for: Ruthenium Incorporated Cobalt Phosphide Nanocubes Derived From a Prussian Blue Analog for Enhanced Hydrogen Evolution
Source: Front Chem. 2018 Oct 30;6:521. doi: 10.3389/fchem.2018.00521 (PMC6218429; doi:10.3389/fchem.2018.00521)
Supplement: Supplementary file 1 [file Data_Sheet_1.docx]

Supplementary Material

Ruthenium Incorporated Cobalt Phosphide Nanocubes Derived from a Prussian Blue Analogue for Hydrogen Evolution

**Yingzhang Yan,^1^ Jinzhen Huang,^3^ Xianjie Wang,^1^ Tangling Gao, ^4,*^ Yumin Zhang,^3^ Tai Yao,^2,*^ and Bo Song^1,2,3,*^**

*** Correspondence:** Tai Yao E-mail: [yaotai@hit.edu.cn](mailto:yaotai@hit.edu.cn); Tangling Gao E-mail: tang5288@163.com;

Bo Song E-mail: [songbo@hit.edu.cn](mailto:songbo@hit.edu.cn)

**1 Turnover frequency calculations**

The turnover frequency (TOF) of the synthesized electrocatalysts was calculated using the following formula ([Kibsgaard, 2015](#_ENREF_5)):

$$\text{TOF=}\frac{\text{\#total hydrogen turn overs/}\text{cm}^{\text{2}}\text{ geometric area}}{\text{\#surface sites / }\text{cm}^{\text{2}}\text{ geometric area}}$$

The total number of hydrogen turnovers:

$$\text{n}_{\text{H}_{\text{2}}}\text{=(j}\frac{\text{mA}}{\text{cm}^{\text{2}}}\text{)}\left( \frac{\text{1C }\text{s}^{\text{-1}}}{\text{1000 }\text{mA}} \right)\left( \frac{\text{1 }\text{mol}\text{ }\text{e}^{\text{-}}}{\text{26485.3C}} \right)\left( \frac{\text{1 }\text{mol}\text{ }\text{H}_{\text{2}}}{\text{2 }\text{mol}\text{ }\text{e}^{\text{-}}} \right)\left( \frac{\text{6.022×}\text{10}^{\text{23}}\text{ }\text{H}_{\text{2}}\text{ }\text{molecules}}{\text{1 }\text{mol}\text{ }\text{H}_{\text{2}}} \right)\text{=3.12×}\text{10}^{\text{15}}\text{ }\frac{\text{H}_{\text{2}}\text{/s}}{\text{cm}^{\text{2}}}\text{ per }\frac{\text{mA}}{\text{cm}^{\text{2}}}$$

The electronic active surface area (ECSA) was calculated according to:

$$\text{A}_{\text{ECSA}}=\frac{\text{C}_{\text{dl}}}{\text{C}_{\text{s}}}$$

*C*_s_ is refer to the specific capacitance which we assume 40 μF cm^-2^ here.

We assuming that Ru-CoPs have the roughly the same surface sites per real surface area with CoP: $\text{surface sites=}\left( \frac{\text{4 atoms/unit cell}}{\text{97.03}\text{Å}^{\text{3}}\text{/ unit }\text{cell}} \right)\text{=1.948×}\text{10}^{\text{15}}\text{ atoms }\text{cm}^{\text{-2}}$

TOFs were calculated according to:

$$TOF=\frac{(3.12\times{10}^{15} \frac{H_{2}/\text{s}}{\mathrm{cm}^{-2}}\mathrm{per}\frac{\mathrm{mA}}{\mathrm{cm}^{-2}})\times|j|}{\text{surface site }\times A_{\text{ECSA}}}$$

the calculation results is provided in Figure S4.

# 2 Supplementary Figures and Tables

**2.1 Figures**

#
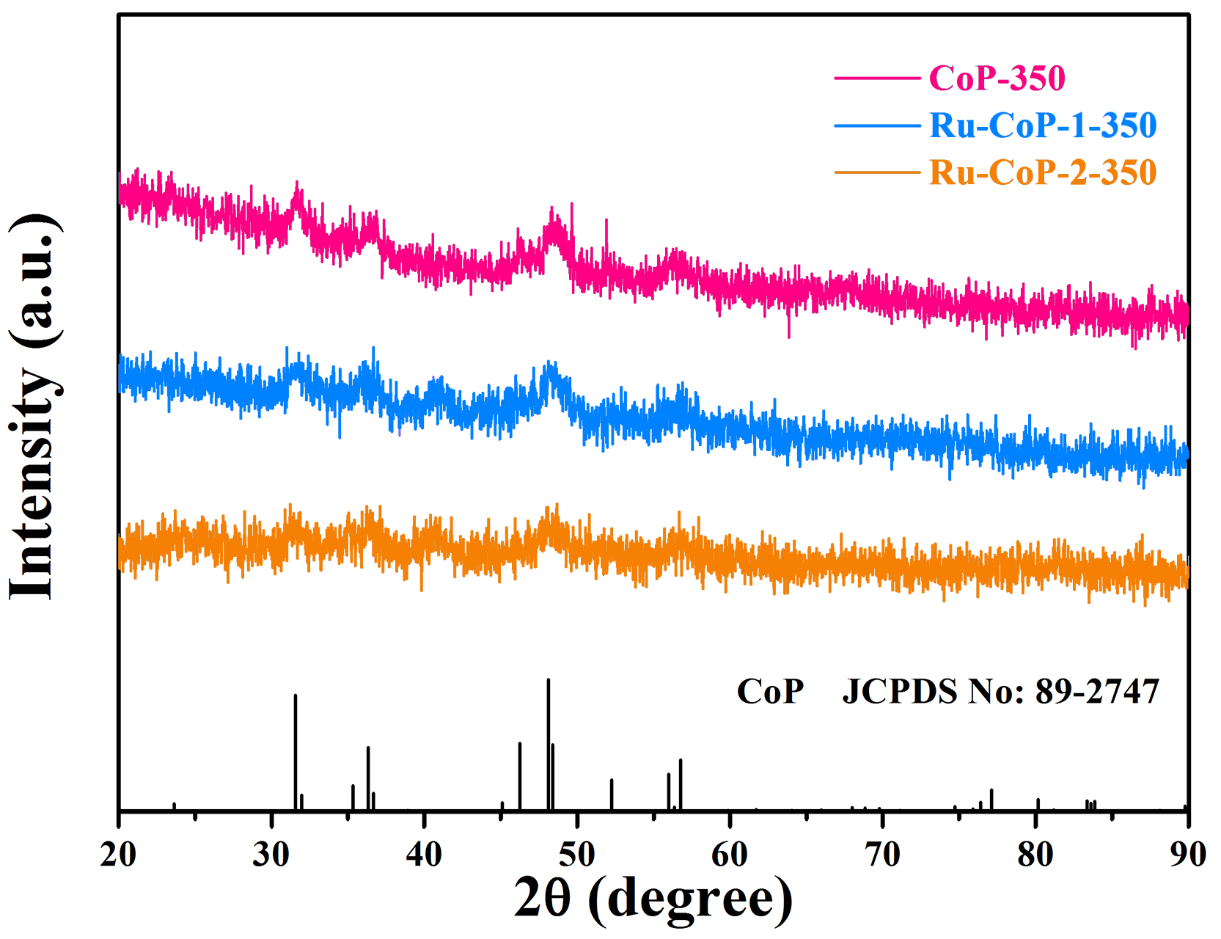


# Supplementary Figure 1. XRD pattern of CoP-350, Ru-CoP-1-350 and Ru-CoP-2-350.


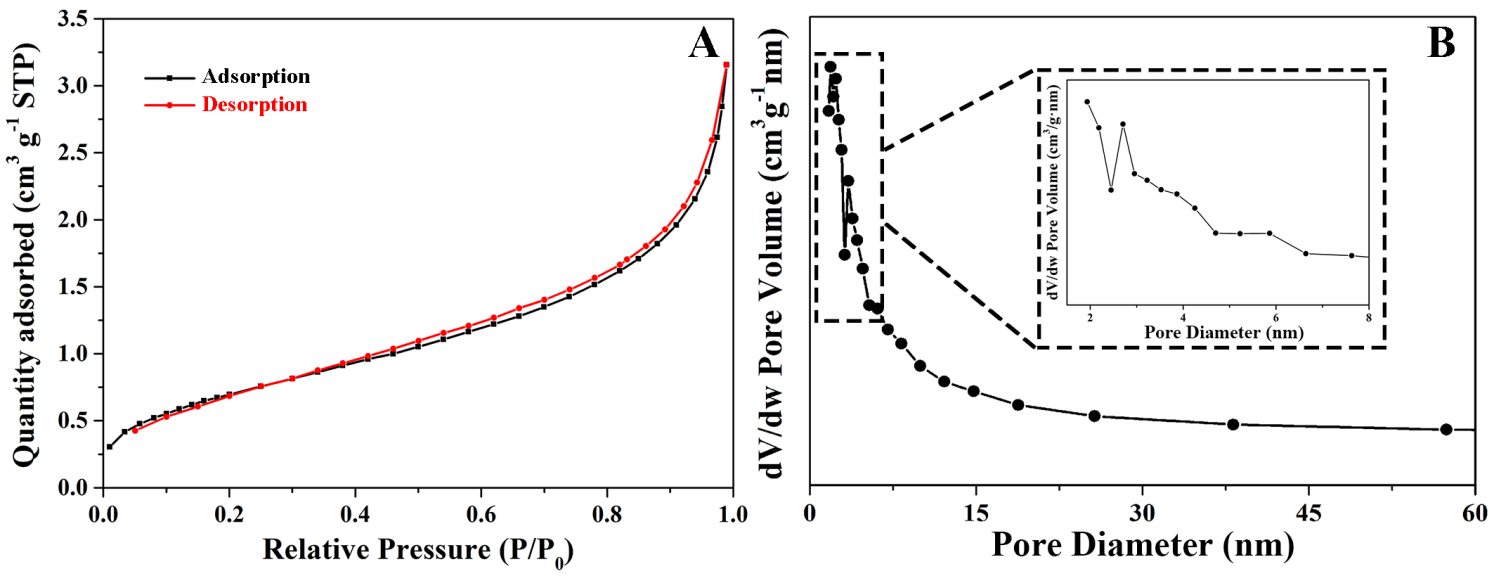


Supplementary Figure 2. (A) N_2_ adsorption–desorption isotherm and (B) pore size distribution plot of Ru-CoP-2-350.


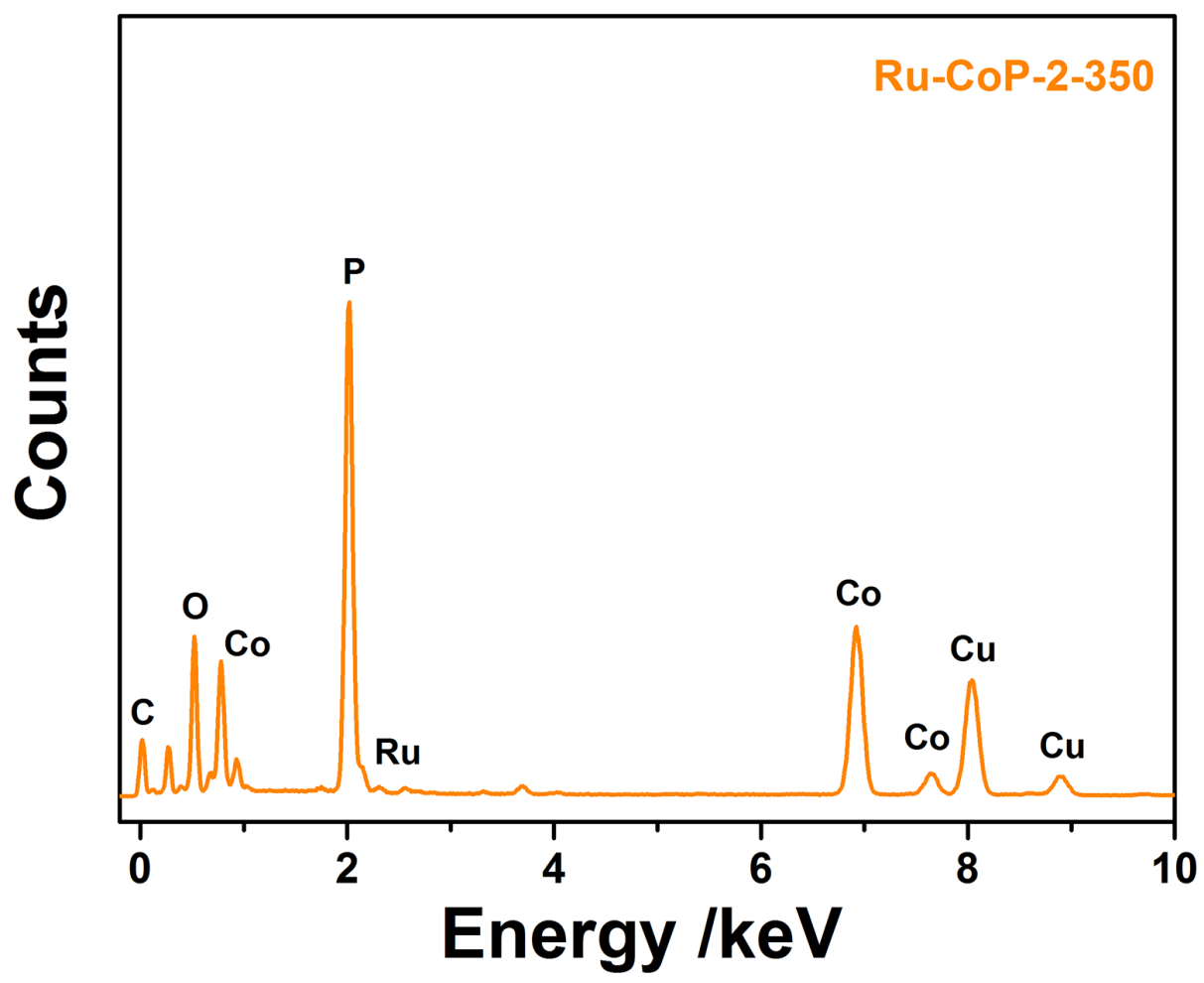


Supplementary Figure 3. EDS data of Ru-CoP-2-350. The Cu is from TEM grid.


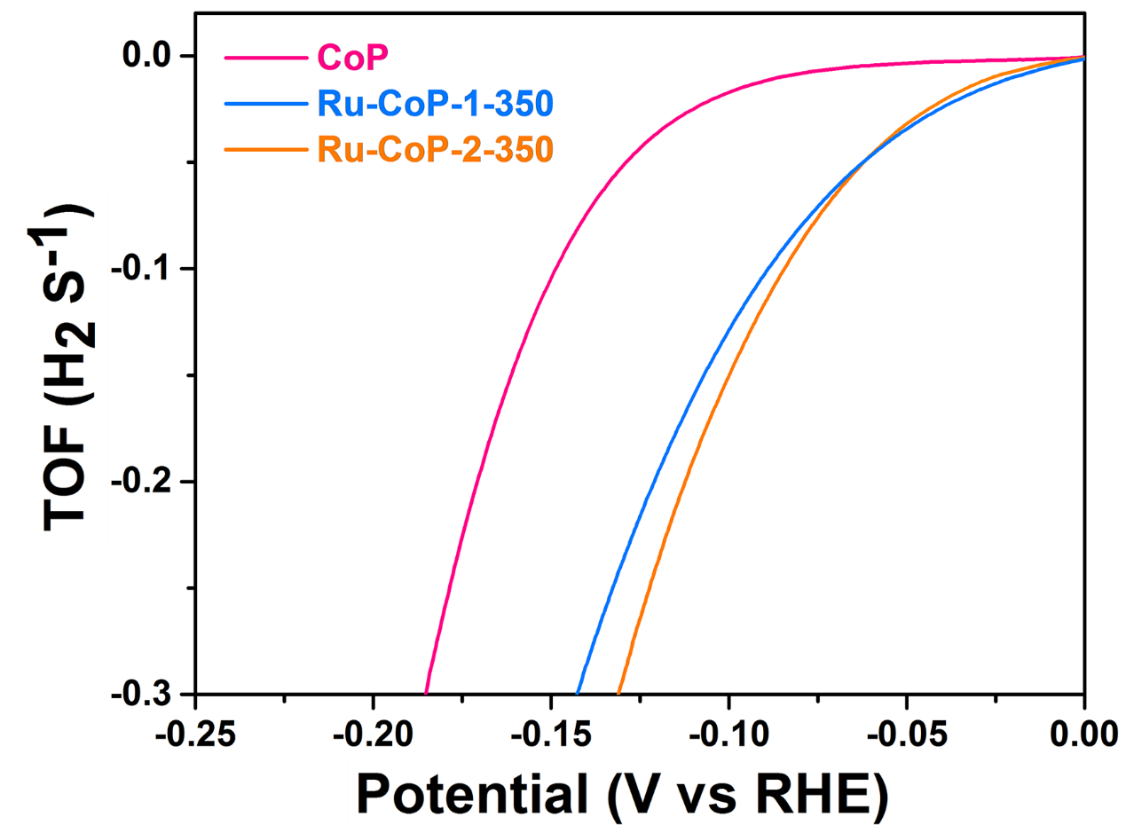


Supplementary Figure 4. TOF calculations of CoP, Ru-CoP-1-350, Ru-CoP-2-350.


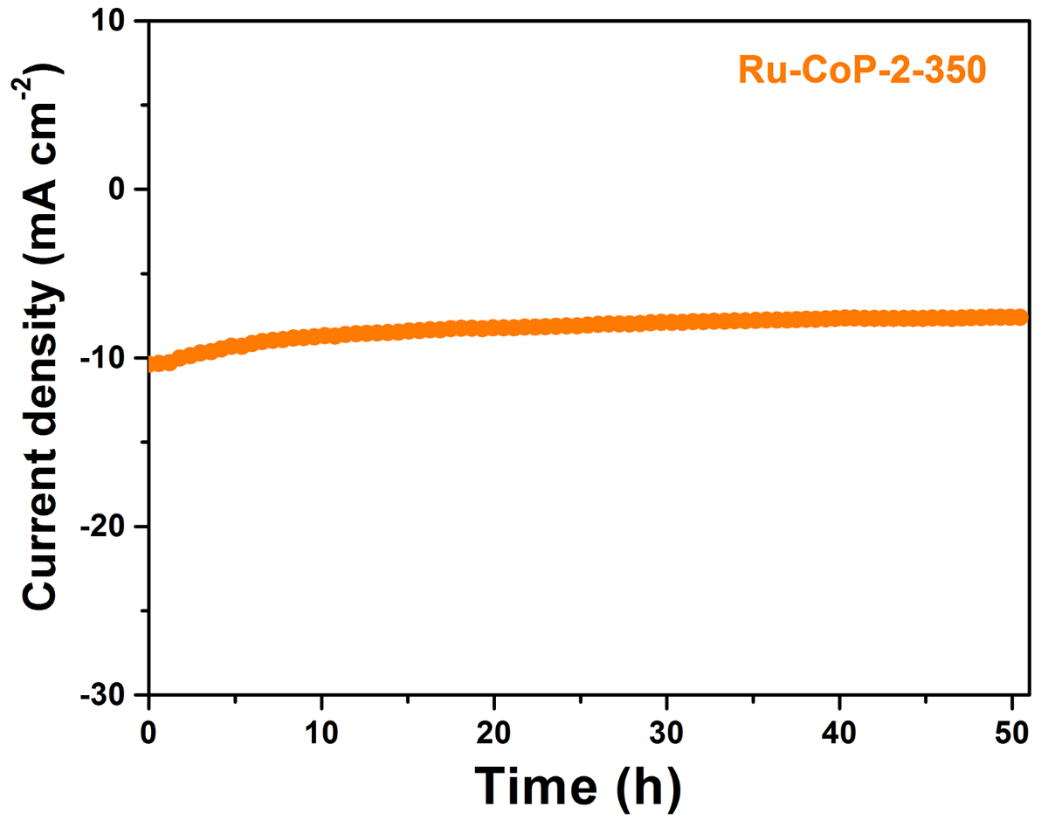


Supplementary Figure 5. Durability test (i-t curve) of Ru-CoP-2-350.


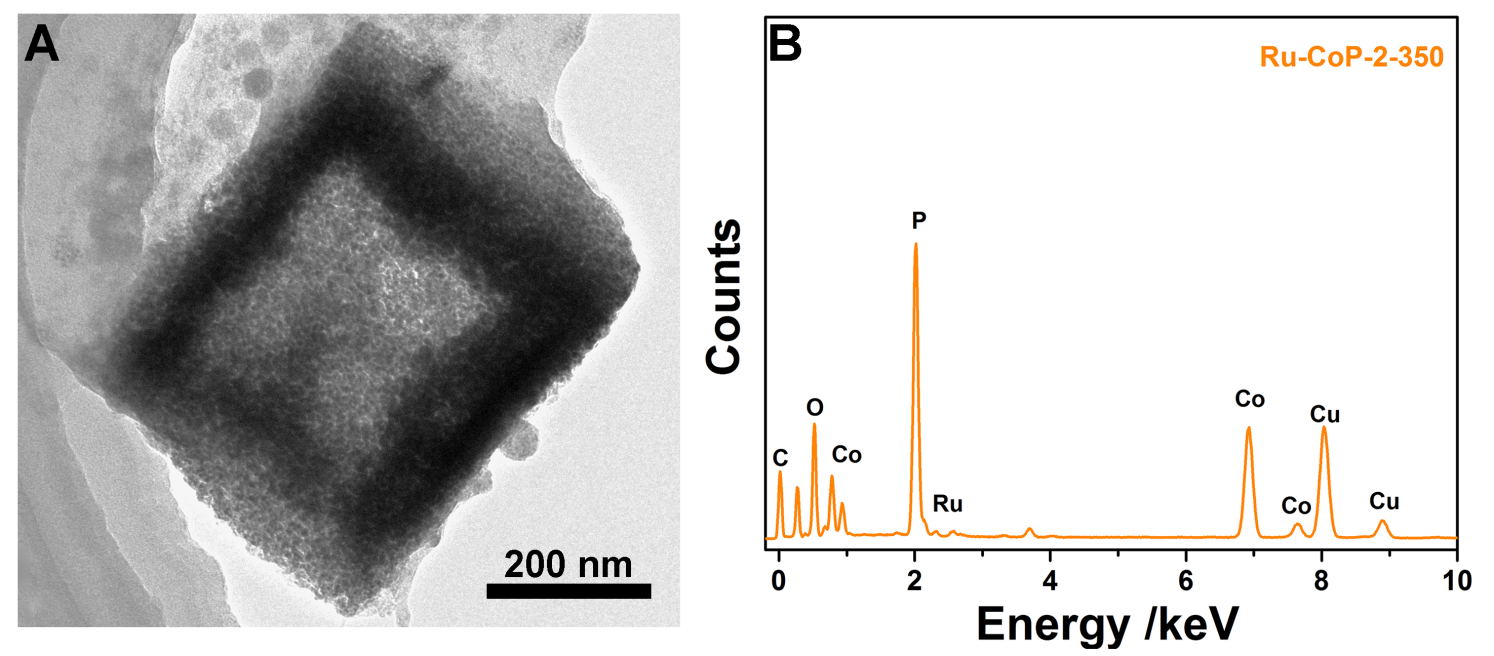


**Supplementary Figure 6.** (A) TEM image of Ru-CoP-2-350 after stability test and (B) EDS of Ru-CoP-2-350 after stability test, the Cu is from TEM grid.

**2.2 Tables**

Table 1 Metal phosphide electrocatalysts for HER in alkaline media (1 M KOH) reported recently (N/A refers to not mention).

| **Catalyst** | **Electrode** | **Loading amount**  **(mg cm^-2^)** | ***η* (mV)@**  **10mA cm^-2^** | **Tafel Slope**  **(mV dec^-1^)** | **Reference** |
| --- | --- | --- | --- | --- | --- |
| **Ru-CoP-2-350** | **GCE** | **0.3** | **51** | **53.8** | **This work** |
| CoP_2_@rGO | GCE | 0.285 | 88 | 50 | ([Zhou et al., 2016](#_ENREF_9)) |
| CoP/rGO-400 | GCE | 0.28 | 150 | 38 | ([Jiao et al., 2016](#_ENREF_4)) |
| CoCeP | Ti | N/A | 98 | 70.3 | ([Gao et al., 2017](#_ENREF_2)) |
| NiFeP | GCE | N/A | 182 | 85 | ([Xuan et al., 2017](#_ENREF_8)) |
| RuP_2_@NPC | GCE | N/A | 52 | 69 | ([Pu et al., 2017](#_ENREF_7)) |
| FeP/GA | GCE | N/A | 240 | N/A | ([Alam Venugopal et al., 2018](#_ENREF_1)) |
| CoP NA/CC | Carbon cloth | N/A | 52 | N/A | ([Liu et al., 2017](#_ENREF_6)) |
| Np-CoP_3_ | Ti mesh | N/A | 76 | N/A | ([Ji et al., 2018](#_ENREF_3)) |

**Reference**

Alam Venugopal, N.K., Yin, S., Li, Y., Xue, H., Xu, Y., Li, X., et al. (2018). Prussian Blue Derived FeP Nanoparticles in Porous Graphene Aerogel as Efficient Electrocatalysts for Hydrogen Evolution Reaction. *Chem Asian J* 13(6), 679-685.

Gao, W., Yan, M., Cheung, H.Y., Xia, Z., Zhou, X., Qin, Y., et al. (2017). Modulating electronic structure of CoP electrocatalysts towards enhanced hydrogen evolution by Ce chemical doping in both acidic and basic media. *Nano Energy* 38,290-296.

Ji, Y., Yang, L., Ren, X., Cui, G., Xiong, X., and Sun, X. (2018). Nanoporous CoP3 Nanowire Array: Acid Etching Preparation and Application as a Highly Active Electrocatalyst for the Hydrogen Evolution Reaction in Alkaline Solution. *ACS Sustainable Chemistry & Engineering* 6(9)**,** 11186-11189.

Jiao, L., Zhou, Y.X., and Jiang, H.L. (2016). Metal-Organic Framework-Based CoP/Reduced Graphene Oxide: High-Performance Bifunctional Electrocatalyst for Overall Water Splitting. *Chemical Science* 7(3)**,** 1690-1695.

Kibsgaard, J. (2015). Designing an improved transition metal phosphide catalyst for hydrogen evolution using experimental and theoretical trends. *Energy & Environmental Science* 8(10)**,** 3022-3029.

Liu, T., Xie, L., Yang, J., Kong, R., Du, G., Asiri, A.M., et al. (2017). Self‐Standing CoP Nanosheets Array: A Three‐Dimensional Bifunctional Catalyst Electrode for Overall Water Splitting in both Neutral and Alkaline Media. *Chemelectrochem* 4(8), 1840-1845.

Pu, Z., Amiinu, I.S., Kou, Z., Li, W., and Mu, S. (2017). RuP2‐Based Catalysts with Platinum‐like Activity and Higher Durability for the Hydrogen Evolution Reaction at All pH Values. *Angew Chem Int Ed Engl* 56(38)**,** 11559-11564.

Xuan, C., Jie, W., Xia, W., Peng, Z., Wu, Z., Wen, L., et al. (2017). Porous Structured Ni–Fe–P Nanocubes Derived from a Prussian Blue Analogue as an Electrocatalyst for Efficient Overall Water Splitting. *Acs Applied Materials & Interfaces* 9(31)**,** 26134-26142.

Zhou, D., He, L., Zhu, W., Hou, X., Wang, K., Du, G., et al. (2016). Interconnected urchin-like cobalt phosphide microspheres film for highly efficient electrochemical hydrogen evolution in both acidic and basic media. *Journal of Materials Chemistry A* 4(26), 10114-10117.
